# Participants’ experiences and impressions of a group-based positive psychology intervention programme for rural adults in Ghana

**DOI:** 10.1080/17482631.2021.1891760

**Published:** 2021-02-28

**Authors:** Richard Appiah, Angelina Wilson Fadiji, Marie P. Wissing, Lusilda Schutte

**Affiliations:** a Africa Unit for Transdisciplinary Health Research, Faculty of Health Sciences, North-West University, Potchefstroom, South Africa; b College of Health Sciences, University of Ghana, Accra, Ghana; c Department of Educational Psychology, Faculty of Education, University of Pretoria, Pretoria, South Africa

**Keywords:** Positive psychology intervention, participants’ experiences, mental health, rural poor adults, qualitative study, inspired life programme, Ghana

## Abstract

**Introduction**: There is growing evidence that group-based mental health intervention programmes can encourage the development of peer support, psychosocial skills, and collaborative therapeutic relationships with longer lasting effects. This study explored participants’ experiences of, perceived benefits of, and recommendations to improve a 10-session group-based multicomponent positive psychology intervention (mPPI)—the *Inspired Life Programme* (ILP)—designed to promote positive mental health and reduce symptoms of depression and negative affect in a sample of rural Ghanaian adults.

**Method**: Face-to-face semi-structured individual interviews were conducted with 18 randomly selected programme participants three months after their participation in the ILP. Data were analysed thematically with an inductive approach.

**Results**: Participants described their experience of the ILP as a forum for growth that granted them the opportunity to introspect, practicalise and situate everyday life challenges, connect with others, and to develop a sense of mutual accountability. Results indicate that the ILP  led participants to develop a stronger sense of positivity and well-being, fructify their ideas, and to cultivate stronger social networks and relationships that led to increased vocational productiveness. Participants recommended that researchers include facets of physical health promotion in the programme and invite close relations of participants to participate in the programme.

**Conclusion**: This study provides the first insight into participants’ experiences of a group-based mPPI in Ghana. These findings may provide useful information to inform the design of context-appropriate community-based mental health interventions to fit participants’ specific needs, capacities, and circumstances.

## Introduction

The past decade has seen the rapid growth in mental health research and interventions designed to promote the well-being of individuals, communities, and institutions (Bolier et al., 2013; Hendriks et al., 2019; Schotanus-Dijkstra et al., 2019; Weiss et al., 2016). Recent research efforts have led to a gradual shift from the biomedical model (Engel, 1977), which focuses on diagnosing and treating physical and mental illness, to a more expansive approach that also explores and promotes the well-being and mental health of the general, non-clinical population (Keyes, 2005; Keyes & Martin, 2017). Although a significant number of people present with mental disorders at the global level (Gil-Rivas et al., 2019), the majority of individuals exhibit moderate mental health, in the absence of psychopathological symptoms (Keyes, 2005; Westerhof & Keyes, 2010; Wissing & Temane, 2013). Furthermore, evidence suggests that the absence of mental illness does not necessarily imply the presence of positive mental health. In this regard, according to the dual-continua model (see Keyes, 2005; Westerhof & Keyes, 2010), mental health is conceptualised to include a state wherein individuals exhibit both the presence of positive mental health as well as the absence of mental illness, rather than just the absence of psychopathological symptoms.

Decades of research have resulted in empirically-driven frameworks that translate mental health and well-being theories and research into positive psychology interventions (PPIs) to facilitate the promotion of positive human development. For instance, PPIs have been developed that promote flourishing by expressing gratitude (Cunha et al., 2019), counting blessings (Deng et al., 2019), savouring (appreciating positive experiences and regulating positive feelings; Smith & Hanni, 2019), using personal strengths (Van Woerkom & Meyers, 2019), practicing kindness (Rowland & Curry, 2018), or setting meaningful goals (Bruhn et al., 2016). A growing body of evidence suggests that PPIs can increase emotional and psychological well-being (Bolier et al., 2013; Hendriks et al., 2019; Weiss et al., 2016). Although several PPI evaluation studies report substantial gains in overall mental health (see Hendriks et al., 2019; Page & Vella-Brodrick, 2013; Ruini & Ryff, 2016; Weiss et al., 2016) or specific dimensions of mental health (e.g., Chakhssi et al., 2018; Kaplan et al., 2014), there is limited literature that explores the individual experiences of taking part in group-based intervention programmes at the community level, particularly in the sub-Saharan African context. Of note, only a few studies also examine the processes and factors that facilitate the effectiveness of group-based mental health and well-being interventions (e.g., Kerkelä et al., 2015; McArdle et al., 2012; Mitchell et al., 2018), particularly in the African context.

The majority of studies that evaluate the effectiveness of interventions apply a randomized controlled trial (RCT) design (Bolier et al., 2013; Hendriks et al., 2019; Weiss et al., 2016). Although the RCT design is considered the gold standard in clinical research which may result in high-quality data that permits the description of causal relationships (Hariton & Locascio, 2018; Spieth et al., 2016), the design has been critiqued in the psychological and social domains, particularly due to its limitations in clarifying the processes that lead to the evidence that it demonstrates (see American Psychological Association, 2006; Bohlin & Sager, 2011). The majority of PPIs in the African context evaluate the effect of the implemented interventions mainly by quantitative methodology and with samples predominantly from South Africa who were mostly drawn from urban and educational settings (e.g., Bach & Guse, 2015; Pretorius et al., 2008; Van Zyl & Rothmann, 2012). In Ghana, there is paucity of research that evaluates PPIs in any population. In a recent collaborative effort, Appiah, Wilson-Fadiji et al. (2020) formulated and evaluated the effects of a 10-session multicomponent PPI (mPPI; the *Inspired Life Programme* [ILP]) in promoting mental health and decreasing symptoms of depression and negative affect among a sample of rural poor adults in Ghana, within a quasi-randomized controlled trial design. The effectiveness of the intervention programme was assessed with a battery of translated and validated Twi versions of mental health and well-being questionnaires in Ghana (see Appiah, Schutte et al., 2020). The results showed moderate to large positive effect of the intervention on mental health three months post-intervention in the experimental group, relative to the control group.

While there exist studies in other settings that employ qualitative methods in their evaluation of PPIs, the majority report on related factors of the intervention, such as the levels of engagement or participation and perceived benefits of the intervention. For instance, Ilvig et al. (2018) explored the significance of an Acceptance and Commitment Theory-based programme on the everyday-life activities of a sample of vulnerable adult citizens in a municipality in the region of Southern Denmark. The programme was designed to motivate participants to improve their well-being, to increase participants’ awareness of the factors that limit their participation in everyday-life activities, and to translate the awareness of these factors to facilitate their daily activities. The findings, based on participants’ accounts of their experiences, showed that the programme enabled them to cultivate social skills and trust, which contributed to increasing their self-confidence and fostering new competencies and courage. In another study, researchers examined the perceived benefits of participating in a strengths-based “Three Good Things” intervention, which was designed to strengthen participants’ skills to deal with stressful events, in a sample of American neonatal healthcare professionals (Rippstein-Leuenberger et al., 2017). Participants expressed that the intervention supported them to cultivate stronger supportive relationships, improve their sense of well-being and happiness, and make meaningful use of their time.

Empirical evidence suggests that exploring the subjective experiences of participants involved in an intervention contribute greatly to understanding the processes and factors that facilitate the therapeutic effect of the intervention (Kerkelä et al., 2015; Mitchell et al., 2018). This bottom-up approach also allows researchers to gain in-depth insights into the underlying thoughts, feelings, and motivations associated with participants’ emotional and behavioural changes during and after the intervention programme (Kerkelä et al., 2015). Considering that participants’ experiential accounts of a phenomenon increase our understanding of the phenomenon, findings from qualitative studies that explore participants’ experiences of interventions programmes can provide useful information for researchers to improve on similar locally-tailored intervention programmes.

### The present study

The aims of the present study were to (1) explore the general experience of individuals who participated in the ILP intervention programme at three months post-intervention, (2) explore participants’ perceived benefits of participating in the ILP intervention programme at three months post-intervention, and (3) solicit for participants’ recommendations to improve the ILP. Findings from this study will provide insight into how people from a collectivistic, rural poor context (who are mostly underrepresented in the literature) experienced a group-based programme aimed at promoting positive mental health in the African context. Additionally, the findings may be used to improve on similar group-based intervention programmes to meet individuals’ specific needs and circumstances.

## Method

We followed the guidelines specified by the COnsolidated criteria for REporting Qualitative research checklist (COREQ; Tong et al., 2007) to report on aspects of the research team, study methods, context of the study, analysis, findings, and interpretation of the findings, which are in line with recommendations by Levitt et al. (2017) to promote methodological integrity in qualitative research methods.

### The research team

All authors have training in psychology, health promotion, and qualitative research methodology and have previously conducted research on mental health and behavioural change interventions with samples drawn from urban and rural community settings.

### Study design

We adopted an exploratory descriptive qualitative research design (Creswell, 2014) to comprehensively explore and describe participants’ experiences of, perceived benefits of, and recommendations to improve the ILP three months after participating in the intervention programme. Qualitative research is appropriate for understanding the experiences of individuals regarding a particular phenomenon or activity (Creswell, 2014; Hammarberg et al., 2016).

### Setting and participants

This study was conducted in four rural poor communities in the Sunyani West District of the Brong Ahafo region of Ghana. The majority of residents of these communities were classified as poor with individual earnings ranging from US$ 1.25 or less aday (categorized as ultra-poor) to US$1.90 aday (classified as poor; Ghana Statistical Service et al., 2015). Almost all residents of all four communities are small-scale farmers who trade in farm produce and share similar socioeconomic characteristics (Ghana Statistical Service et al., 2015). The majority of residents are middle-aged, Christians, live in a collectivistic social setting, and had a basic school level of education (Ghana Statistical Service et al., 2015).

We randomly selected a subset of individuals who were previously drawn from four rural poor communities to participate in a two-hour, once-weekly, 10-session mPPI (see Appiah, Wilson-Fadiji, et al., 2020). At three months post-intervention, participants for this study were randomly selected from a list that comprised of all individuals in the experimental group (*n* = 40) who participated in the mPPI, by means of a computer-generated number sequence created with Excel. Participants were recruited and interviewed, sequentially, until data saturation was reached with the 18th participant. One participant relocated out of the community and could not be contacted, and therefore the next randomly generated participant number was used to identify the next participant to be included. The majority of the participants in the present study were females, married, and had a primary level or no formal education. Participants were aged between 19 and 58 years (*M* = 34.4, *SD* = 10.2). Table I summarizes the characteristics of the sample.

**Table I.** Sample characteristics (*N* = 18)

Characteristics		*n* or (*M* ± *SD*; range)
Age (years)		34.4 ± 10.2; 19–58
Gender		
	Male	8
	Female	10
Marital status		
	Single	3
	Married	11
	Co-habiting	1
	Divorced/Separated	2
	Widowed	1
Educational level		
	None	8
	Primary	5
	Junior high	3
	Secondary	1
	Tertiary	0
	Other	1

*M* = Mean; *SD* = Standard deviation.

### The ILP intervention programme

The 10-session mPPI was formulated using a community-based participatory research approach. The formulation of the ILP intervention involved a seven-step process, namely, reviewing existing theory and programmes; defining the structure, strategy, and content of the intervention; conducting community consultation and cognitive-appraisals; simulation of sessions; experts’ review; pilot-testing of manualised sessions; and review of final sessions. The ILP is based primarily on selected constructs and principles of positive psychology that have been found to promote and protect mental health, including self-acceptance (Page & Vella-Brodrick, 2013; Ruini & Ryff, 2016), meaning and purpose in life (Martela & Steger, 2016), personal growth (Ruini & Ryff, 2016), kindness (Curry et al., 2018), empathy (Hodges et al., 2011), and positive relationships with others (Proyer et al., 2015). These constructs were integrated with four other constructs and principles underlying Beck’s cognitive-behaviour intervention model (Beck, 2011), comprising problem solving skills, time management, goal setting skills, and cognitive restructuring, to formulate the ILP. Constructs and principles from positive psychology and Beck’s model have been incorporated into several well-being interventions to promote mental health in various contexts (see Damreihani et al., 2018; Ryff, 2014; Smith & Hanni, 2019; Weiss et al., 2016). Overall, the ILP was designed to promote purposeful and meaningful life, optimism, self-acceptance, personal growth, goal-setting skills, problem-solving skills, cognitive restructuring, and positive thinking patterns through interactive group discussions and activities.

All sessions were facilitated by trained psychology graduates in Twi, which is the most widely spoken Ghanaian language and the native dialect of participants and facilitators. The intervention was supervised by the first author, who is also a native Twi-speaker and a registered psychologist with training in qualitative research. Session facilitators did not participate in sample selection, group assignment, or assessment of participants. All sessions were designed to stimulate interactive discussions where participants take turns to share their views and ask questions. Each session consisted of three main parts: first, a review of the previous session and a discussion of homework assignments from the previous session; second, a discussion of the theme and contents of the current session and a breakout activity; and third, an overview of key lessons, review of the session, and discussion of homework assignments. Sessions included plenary and breakout components where facilitators guided participants through exercises to master specific skills.

### Data collection instrument and procedure

The research team developed a semi-structured interview guide based on the study objectives as well as insights from previous empirical studies that evaluated participants’ experiences of group-based mental health (well-being) interventions (e.g., Ilvig et al., 2018; McArdle et al., 2012; Mitchell et al., 2018; Rippstein-Leuenberger et al., 2017). Prior to participant recruitment, the semi-structured interview guide was pilot-tested with a small sample of seven participants drawn from the target population, and refined accordingly. Led by an independent mediator, the research team conducted home visits to obtain informed consent and recruit participants for the interview. For the main study, as was also the case in the pilot study, each participant was asked three broad initial questions: *“Please tell me about your general experience of the training programme?”, “In what ways, in your view, has the programme benefited you or your community?”*, and *“How can the researchers improve the programme to increase its benefits for future participants?”*. Probes were used to ensure full understanding of participants’ responses and to encourage participants to provide detailed feedback. The 40-minuteface-to-face interviews were conducted by two trained interviewers at participants’ homes at a time convenient to them and supervised by the first author. All interviews were conducted in Twi and audio-taped with participants’ permission.

### Ethical considerations

The study was approved by two institutional review boards: The Health Research Ethics Committee of the North-West University (NWU-00109-17-S1), South Africa, and the Noguchi Memorial Institute for Medical Research Institutioional Review Board of the University of Ghana (NMIMR.IRB CPN 007/17-18), Ghana. Permissions were also obtained from the Regional and District Health Directorates of the SWD and the chief and elders of each community involved, prior to the commencement of the study. The services of a clinical psychologist were made available in the event that a participant experienced any intense negative feelings or thoughts during the interviews, or during group discussions at sessions, such as when identifying or challenging unhelpful/negative thoughts. The processes instituted to ensure confidentiality and anonymity of data and the participant’s right to decline or withdraw from the study, at any time, without any consequences, were fully explained to each participant. Participation in the study was voluntary and written informed consent was obtained from each individual prior to the interviews. Participants who could not sign the consent form were assisted to thumbprint. Efforts instituted to anonymise and secure data on a password protected computer and secured online servers were fully explained to participants. Each participant was presented with a gift (either a bar of soap, packet of sugar, or canned drink) each worth GHS 5 (≈ USD 1) at the end of the interview as a token of appreciation for their participation. A participant who was repeatedly emotional in her description of the challenges and stress in her daily life was referred to the psychologist for support.

### Data analysis

We followed the dynamic equivalence approach (Jin & Nida, 2006) to translate the data from Twi to English, where the focus was on statements with equivalent meaning, rather than word-to-word English translations. Two research associates with bilingual (Twi-English) competence were recruited and trained to translate the data. The first author, who is a native Twi-speaker, provided an example of a translated transcript from a participant’s data. For specific terms and concepts, the translators consulted with the first author for clarification. The first author and another research associate cross-checked the translated scripts with the audio data to ensure that vocabularies and concepts in the translated text represented participants’ expressions and corresponded with the context of the study.

We followed the inductive thematic analysis framework (Braun & Clarke, 2012) to systematically analyse the data generated for each of the three aims. Thematic analysis uses a systematic approach to identify patterns across a dataset, enabling a rich and detailed analysis of participants’ perspectives (Braun & Clarke, 2012). First, the first author and an independent co-coder read the text (generated by each specific question) several times carefully to understand participants’ narratives and to identify texts that were meaningful and relevant to each of the three specific aims. Second, all units of text that describe the same content for each aim were identified, grouped, and assigned tentative codes. Third, the dataset generated by each specific question for each specific aim was systematically reviewed to validate the names, definitions, and quotes assigned to the emerged themes. The two research associates who transcribed the data independently verified the codes and themes to ensure their replicability and coherence. The first author and the research associates reviewed the analytical approach and process and held discussions to reach consensus on each emerged codes and themes.

### Trustworthiness

Trustworthiness was ensured by applying the principles of credibility, dependability, confirmability, and transferability (Guba & Lincoln, 1989; Lincoln & Guba, 1985) as well as concepts of fidelity to the subject matter and utility in achieving research goals postulated by Levitt et al. (2017). First, to increase the credibility of the data, member checking was done by engaging participants to clarify and confirm interpretations or understandings inferred from their accounts during the interview process, on-spot, to ensure that the correct interpretation was derived from participants’ responses (Guba & Lincoln, 1989). Second, to ensure transferability, a detailed description of the stages of the research process has been provided for clarity and to allow replication of the study in a similar setting. Records of the raw data, field notes, and transcripts were systemised and cross-referenced to reach the reported conclusion in order to increase dependability of study findings and to improve the fidelity of the subject matter (Levitt et al., 2017). Verbatim quotes were used to report findings. Furthermore, the research team conducted peer debriefing to discuss the emerged themes to ensure that all aspects of the data was covered. The rationale and justifications underpinning the methodological and analytical choices of the study are explicitly described to enhance confirmability (Koch, 1994; Lincoln & Guba, 1985) and to increase the utility in achieving the study goals (Levitt et al., 2017).

The first author played a lead role in the conceptualisation, design, implementation, and the main programme evaluation of the ILP, which is reported elsewhere (see Appiah, Wilson-Fadiji, et al., 2020). The researcher made conscious efforts to identify and acknowledge all potential personal (and analytical) biases at all stages of the research process. To minimize biases and as a means to increase quality assurance (see Braun & Clarke, 2012; Tong et al., 2007), a reflexive diary was maintained where reflexive commentaries were made and reflected on.

## Results

The study identified nine themes—four, three, and two themes for the first, second, and third aims, respectively—that together describe participants’ experience of the ILP intervention programme, their perceived benefits of the ILP, and their recommendations to improve the ILP intervention programme. Table II presents a summary of the aims of the study and the emerged themes. For the first aim, four themes (*Self-reflection; Practicality and relatability of programme; Mutual engagements and self-disclosure;* and *Sense of responsibility and accountability*) emerged that together describe participants’ experiences of the ILP intervention programme. Three themes were identified that represent participants’ perceived benefits of the ILP intervention programme (i.e., *Improved sense of well-being and happiness; Enhanced social networks and relationships;* and *Increased vocational productiveness and goal achievement*) for the second aim. Lastly, two themes also emerged (i.e., *Include facets of physical health promotion* and I*nclude close others in intervention*) that explicate participants’ recommendations to improve the intervention programme.

**Table II.** Summary of the study aims and emerged themes

Study aims	Themes
Explore participants’ experience of the ILP intervention programme	1.1 Self-reflection 1.2 Practicality and relatability of programme; 1.3 Mutual engagements and self-disclosure 1.4 Sense of responsibility and accountability
2. Explore participants’ impression of the ILP intervention programme	2.1 Improved sense of well-being and happiness 2.2 Enhanced social networks and relationships 2.3 Increased vocational productiveness and goal attainment
3. Solicit for participants’ recommendations to improve the ILP intervention programme	3.1 Include facets of physical health promotion 3.2 Include close others in intervention

### Aim 1: exploration of participants’ experiences of the ILP

The group-based interactive discussions and skills demonstration approach adopted for the intervention sessions provided a distinctive experiential learning environment which motivated each participant to take a deep reflection of their lives, fructify their ideas, develop mutually beneficial relationships, and inspire a sense of responsibility and accountability.

#### Self-reflection

The majority of the participants described their experience of the ILP intervention programme as a unique learning forum that afforded them the opportunity for careful introspection by evaluating their ideas, thoughts, and feelings about themselves and other close relations. Many of the participants reported that the topics discussed and the lessons shared at the sessions created solemn moments for self-reflection where they took a holistic view of their lives. The breakout sessions, in particular, also impelled most participants to reflect and to deliberate on how they can incorporate these lessons into their daily activities to enhance their overall sense of well-being.

…for me, the things we discussed helped me to take a critical look at my life again … I see that I can live a happy life when I assess my ideas and actions and then act in ways that can make me live a happier and meaningful life … (P5, a 44-year old male)

Participants also reported that the case examples discussed by the session facilitators and the personal experiences shared by members at the sessions stimulated and compelled them to reflect on how they will respond when confronted with similar life circumstances.

… we took turns to share our personal experiences when a topic or issue was discussed … I learned a lot from the personal experiences that were shared … they made me to think through my own issues … (P15, a 38-year old female)

#### Practicality and relatability of programme

Participants recounted how the sessional themes discussed at the sessions fit into their lifestyles and were therefore easy to understand, implement, and sustain. Each session included a practical demonstration component that involved a skill demonstration, which is first led by the facilitators and thereafter by the participants in breakout sessions. Largely, the implementation strategy of the ILP facilitated participants’ efforts in translating the lessons from the sessions into practicable guidelines that they can easily relate with and apply to their life situations.

… all our discussions were very important and were about things that affect our daily lives … they were also very practical … you examine your situation and set your own goals, identify your own strengths and weaknesses … (P2, a 36 year-old male)

… I really liked the discussions because we were talking about the things that were happening in our lives. It was as if our teachers were also staying in the community with us … (P10, a 43-year old male)

#### Mutual engagements and self-disclosure

A major theme that describes participants’ experiences of the intervention programme was the feeling of connectedness and responsibility for each other that were fostered at the sessions. The facilitators commenced each session with a discussion of the potential benefits of attending and participating in the discussions and activities and how these contributions can boost the gains at the collective and individual levels. To this end, the majority of participants actively participated in all sessional discussions and activities, knowing that their presence and contributions were essential to the progress and the effectiveness of the sessions.

… again, all of us knew that our attendance and contributions were very important for the success of the programme … we all promised to attend all the sessions and share our views so we can all learn from each other … (P4, a 32-year old female)

The majority of participants also acknowledged that a number of factors, such as the courteousness of the facilitators, encouraged them to open up to share their personal experiences and to learn from the examples of other members. All sessions of the ILP were structured to allow participants to take a lead in the discussions, go on breakout sessions where they further collaborate with a partner to master skills, and to take turns to share their thoughts and experiences with the group. Most participants, on their own volition, shared their personal experiences as case examples in their contributions to the group discussions.

… it was like a school … we were all allowed to share our views and personal experiences. It was the first time I shared my experiences with other people … it felt good knowing that other people also shared similar challenges and concerns … (P14, a 48-year old female)

#### Sense of responsibility and accountability

Participants also expressed that the session activities, particularly the homework assignments, instilled a sense of responsibility. Participants were required to discuss homework assignments with two family members or friends who were non-participants, and to share the outcome with the group members at the beginning of the next session. Acknowledging that completing their homework assignments could help them and group members to better understand the lessons that were discussed and maximize their gains, most participants invested more efforts into the homework assignments.

… I also felt that others were depending on my findings … to learn from them so I always make the effort to get it done. (P9, a 48-year old male)

… and knowing that my contributions can help others, I always try to do my homework … now when I am doing something, I think about how other people will also benefit from it or how it can affect others. (P3, a 35-year old female)

### Aim 2: exploration of participants’ perceived benefits of the ILP

Findings elaborate participants’ descriptions of the perceived benefits from participating in the ILP intervention programme. The majority of participants indicated specific psychosocial changes that they attributed to the ILP intervention, namely, improved sense of well-being and happiness, enhanced social networks and relationships, and increased vocational productivity and goal achievement.

#### Improved sense of well-being and happiness

The majority of the participants ascribed the observed positive changes and improvements in their general sense of well-being to the ILP. For most participants, participation in the programme enabled them to acknowledge and accept multiple aspects of themselves (i.e., strengths and weaknesses). Overall, the ILP programme appeared to have provided participants the impetus to develop a positive outlook to life, increased their creativity and constructive cognitions, and improved their overall well-being and happiness.

… I think that I feel happier after the training … now I do not bother so much about situations I cannot change … which can make me feel sad or jealous of other people … I look at the good things in my life … and how to take care of my family. (P18, a 42-year old male)

Some other participants associated the ILP to the positive cognitive and behavioural changes they observed in their lives. More specifically, there were reports of improvement in self-awareness, deeper appreciation of strengths and weaknesses, increased sense of hope and optimism, and an improved sense of well-being as a result of participating in the ILP.

… I used to complain and dislike a lot of things about myself … when I compare myself with other women … but now I recognise and accept my limitations, and I am working hard to achieve more … I think I feel better. (P1, a 38-year old female)

#### Enhanced social networks and relationships

Participants particularly reported that the ILP improved their social engagements and connectedness as a result of the lessons and skills acquired from the programme, and particularly, from participants’ engagement with session facilitators and group members. For other participants, the session on *“My Nourished Relationships”* that explores the features of positive relationships enabled them to identify various ways and actions that support and improve the quality of their relationships.

… one of my goals was to learn to develop better relationship with my husband and his brother. I had a lot of issues with my husband … I learned a lot from the training and I can say it has helped me to relate well with him … now we live peacefully … (P17, a 51-year old female)

The development of new and the strengthening of existing supportive social networks were additional benefits mentioned by a significant number of participants. There were reports of formation of support social groups to work in mutually agreed terms to achieve their self-identified goals, including helping each other on their farms and building or renovating their thatched (mud) houses ahead of the rainy season. For most participants, the recognition that other group members shared similar difficult challenges in their everyday lives bolstered their confidence to open up to discuss their personal challenges and to look for others with similar issues with whom they could work together in a mutual interest.

… my group partner and I met after the training and we decided to help each other to clear the land (farm land) and plant our maize and cassava. Our wives also joined us … we will harvest by the end of August … (P11, a 57-year old male)

#### Increased vocational productiveness and goal attainment

The data showed that participants made several efforts to incorporate the lessons on short- and long-term goal-setting strategies, time management, and problem-solving skills into their daily activities to increase their vocational productivity. The majority of participants reported that the practical discussions and exercise on life skills were helpful in guiding them to translate the lessons from the sessions into formulating, planning, and executing their self-identified goals that were set at the initial stages of the programme.

… now I am able to plan my life well … before I take any action, I first think about the effects and the process that I can follow to achieve it … like my farm … I have now increased it to almost two acres … (P16, a 39-year old female)

Several references were also made to other observed positive behavioural changes that contributed to the increased vocational productivity. For instance, some participants reported becoming more conscious of “*time robbers*”—situations and factors that waste their productive time. Participants recounted that the sessional activities on setting *SMART* goals and effective time management using the *empty jar demonstration* also contributed to the efficient use of their time and resources in recent times.

… the goal setting exercise with the empty containers always reminds me to plan everything I want to do … now I usually plan my day and week ahead … (P13, a 28-year old female)

### Aim 3: recommendations to improve the ILP

Participants made two recommendations for improving the content and delivery of the ILP intervention. The first was the suggestion to include specific physical health-related topics in the intervention programme. The second recommendation was to include close others and relatives of participants in the intervention programme.

#### Include facets of physical health promotion

A recurring recommendation by participants to improve the ILP intervention was that other sessions that discuss aspects of physical health, such as healthy eating, exercises, effects of alcohol and drug abuse, and ways to relax and rejuvenate their aching bodies after tedious farm work should be included to extend the scope of the intervention. Two participants stated,

… maybe next time you should add other topics for us to discuss … other health issues such as proper eating and exercise to stay healthy … and how to control hypertension … (P7, a 54-year old male)

… you can also include some discussions to teach us about ways to stay fit … to manage our stress … and be healthy to take care of our children and farm … (P4, a 32-year old female)

#### Include close others in intervention

It was also suggested that future programmes should extend invitations to family members and friends of participants who show keen interest in the programme to participate. It was frequently reported that individuals, mostly family members and friends, with whom participants discussed homework assignments also expressed profound interest and wished they were included in the programme.

… everyday my wife will ask me to tell her all the things we discussed … she asked me several times if she could be permitted to join in the meetings … it will be good if they are also invited to join so we can all learn together. (P8, a 48-year old male)

… my son and neighbour were also interested and wished they could join … the teachers said only the people selected are allowed … maybe they can consider allowing other people who also desired to attend to join the sessions …. (P6, a 51-year old male)

## Discussion

The present study set out to explore participants’ experiences and impression of the *Inspired Life Programme* (ILP), a multicomponent positive psychology intervention (mPPI) designed to promote positive mental health and reduce symptoms of depression and negative affect in a sample of rural poor adults in Ghana. The aim of the current study was not to evaluate the effectiveness of the intervention in achieving these outcomes (this was investigated in a study that applied a quasi-RCT approach, which is reported in Appiah, Wilson-Fadiji, et al., 2020), but rather to explore participants’ general experience and perceived benefits of the programme, and to solicit participants’ recommendations to improve the ILP. Generally, participants expressed that the ILP inspired them to introspect, fructify their ideas, and form mutually beneficial partnerships. Participants also described that the ILP led to improved sense of well-being, stronger social networks and relationships, and increased vocational productiveness and goal attainment. Participants recommended that the scope of intervention programme can be extended to also include aspects of physical health, and that family members and friends could be invited to also join the sessions. The findings of this study not only allow for a holistic evaluation of participants’ experiences of the programme, but also provide further in-depth understanding of the adjunct quasi-RCT results (see Appiah, Wilson-Fadiji, et al., 2020). It is noteworthy that the programme appeared to affect multiple aspects of well-being by improving positive intrapersonal feelings (sense of well-being and happiness), positive interpersonal skills (social networks and relationships), and positive functioning (vocational productivity). This finding corroborates with previous research that suggests that positive feelings and positive functioning are both markers of well-being (e.g., Kashdan et al., 2008; Keyes, 2005).

The ILP appeared to have encouraged the participants to reflect on and evaluate their thoughts, feelings, and behaviours. On this point, one session of the ILP teaches participants how to recognise their strengths and accept their weaknesses and set their personal growth and development goals. Another session encouraged participants to discuss their values and personal goals in life and engagements that bring meaning to their lives. The discussions and sessional activities were practicalised to fit into participants’ circumstances and daily activities. Our findings corroborate with earlier studies (e.g., Moxham et al., 2017) that suggest that individuals who set and evaluate self-identified goals are more motivated to achieve their goals, which inherently, facilitates resilience building and promotion of mental health, more generally. A possible socio-cultural explanation for this finding could be the prevailing cultural norms and practices in the study context. The Ghanaian culture, and African culture in general, embrace self-reflection and introspection (Kpanake, 2018). The ILP appears to be the first of its kind to afford participants a stimulating group interaction and peer-learning experience that provided opportunities for self-reflection, while also engaging with facilitators and group members to discuss important issues that affected their daily lives. It is possible that these introspective engagements may have provided participants with a clearer understanding of themselves and therefore, a clearer sense of purpose.

PPIs produce better results when they are practicable, convenient, directly relate to participants’ circumstances, and can be easily implemented in participants’ daily lives (Lyubomirsky & Layous, 2013). It was noted that the activities in the ILP were tailored to fit into participants’ lifestyles and were therefore easy to understand, implement, and sustain. This can be attributed to the bottom-up approach adopted in formulating the ILP, where community members were consulted to appraise and make suggestions on the content, structure, and delivery strategies of the sessions. The participants in the ILP valued the practical nature of the sessions and the ease with which they could relate with and incorporate the lessons and skills learned from the sessions into their daily routines. This feature of the intervention carries an important practical implication, considering that people resort to alternative behaviours when new actions do not easily integrate into existing routines (see Lally et al., 2011). It is possible that the inclusion of practical demonstration exercises, such as the empty jar demonstration to teach effective time management, goal-setting skills, and problem-solving skills may have aligned with participants’ life skills needs and thus heightened their interest in the sessions. The theoretical and practical implications of this finding is that the theories and principles of positive psychology and cognitive-behavioural intervention that underpin the ILP could have fostered practical utility and relevance in the Ghanaian rural context and particularly for the current sample, as these approaches have also been used to promote mental health of various groups in other settings (e.g., Bolier et al., 2013; Hendriks et al., 2018; Weiss et al., 2016).

An effective mechanism in the programme that may have contributed to participants’ positive experience of the ILP was the group-based delivery approach, which allowed participants to share their experiences with other group members through interactive discussions and activities. Group interactions, made possible by the collective attendance and participation in session discussions and activities, are important vehicles for initiating and maintaining positive experiences, which can have a positive influence on overall well-being (Borek & Abraham, 2018; Teodorczuk et al., 2019). As observed from the findings, the breakout sessions, where participants paired up to further deliberate on the issues under discussion and for practising skills, created avenues for participants to learn from each other and to recognise and verbalise their feelings. There were also a few participants with high levels of mental energy who inspired group members with their positive contributions, and thereby encouraged others to open up to share their views and personal experiences with the group members. A possible explanation for the high level of group interactivity and mutual engagements in this sample could be the collectivistic cultural orientation and synergism which are typical of the Ghanaian rural setting (see Appiah, 2020; Gyekye, 2013). It was observed that participants willingly took turns to share and cite their personal experiences at the group discussions for other group members to draw lessons from and to serve as example situations when specific themes are being discussed. This level of engagement, in addition to the facilitators’ familiarity with the culture of the participants, might have maintained the high interest and actuated the interactivity and self-disclosure of participants.

The high level of connectedness and sense of responsibility and accountability exhibited by participants at the sessions, and the reported gains thereof, add to the existing evidence that interventions that encourage participants to be accountable and responsible in groups may lead to greater fidelity and mastery of the skill involved (see Cleo et al., 2018). This may have caused participants to feel valued and increased their belief that they mattered because they are adding value to others’ lives (see Prilleltensky, 2019). Nonetheless, it is noted that participants did not report self-accountability although they continued to apply these lessons and skills three months after the completion of the intervention. Furthermore, participants recounted that the deep understanding by the facilitators as well as their respectfulness and courteousness stimulated high interest in and interactivity of the group discussions and activities, thereby expediting the bond and cordiality among participants. A practical implication of this finding is that according participants with respect and engaging them as co-creators of knowledge could increase their reception, engagement, and participation in the intervention.

The perceived benefits of the ILP intervention programme reported by participants, in terms of improving mental well-being and increasing vocational productivity, support existing evidence suggesting that psychological well-being can be increased through intentional activities. The findings of the present study not only corroborate our previously reported quantitative findings (see Appiah, Wilson-Fadiji, et al., 2020), which showed improvements in positive mental health, general self-efficacy, satisfaction with life, and subjective happiness after participation in the ILP intervention programme, but also shed light on the specific growth promoting processes that contributed to these positive outcomes. Our findings also extend the literature on PPIs, which has provided extensive evidence for improvements in well-being and mental health, more generally, but not behavioural changes (see Odou & Vella-Brodrick, 2013; Pietrowsky & Mikutta, 2012; Sin & Lyubomirsky, 2009), partly due to the lack of qualitative research to explore the factors and mechanisms underlying the effectiveness of these interventions. In addition to the empirical evidence of the theories and principles underlying the ILP, the deprived socio-economic circumstances of participants may have expedited their interest in the pursuit of practical methods to solve their individual and collective problems—which the ILP intervention was designed for. It is also possible that the pilot-test phase of the ILP development, where community members appraised and contributed to refining the sessional themes and case examples, contributed to the practicality of the intervention sessions and relatability of the sessions to participants’ circumstances and needs.

There appears to be a link between participation in the ILP and social connectedness, which was evidenced in the participants’ narratives of the supportive social networks and the strength of social relationships that have been nurtured among group members. The ILP reportedly encouraged participants to nurture meaningful connections with close family members, friends, and other community members to work together on issues of common interest. This finding aligns with the peer selection model, where individuals seek out friendships with activity levels and goals similar to their own (see Sawka et al., 2013). Our findings also add to existing literature that suggest that interventions designed to foster social inclusion and to promote social relationships can enhance psychological well-being and mental health, more generally (see Tough et al., 2017). A conceptual implication of this finding is that individuals living in collectivistic African settings are more likely to form mutually beneficial partnerships at the individual and group levels to work to resolve identified problems of mutual interest than participants from individualistic cultural orientation (cf. Darwish & Huber, 2003). Research evidence suggests that Ghanaians are generally resilient, supportive of each other, and demonstrate high social connectedness (Abukari, 2018), largely due to the communal societal orientation.

The findings of this study lend some additional weight to evidence suggesting that psychosocial training in life skills, such as goal setting, effective time management, and problem-solving skills can translate into increased vocational productivity (e.g., Clark et al., 2017; Goerg, 2015). Specifically, the present study supports and extends past research in showing that striving for self-concordant goals, venturing into productive activities, and pursuing personal growth could lead to gains in positive mental health and increased productivity (Page & Vella-Brodrick, 2013). There is evidence to suggest that interventions that target mental health lead to improved economic outcomes as well, thus yielding a cycle of increasing returns (see Skeen et al., 2010). Such studies have recommended and advocated for the integration of mental health interventions into social interventions and economic empowerment programmes in developing countries. Considering the large body of evidence that suggests that poor mental health and poverty interact in a negative cycle in low- and middle-income countries (see Patel et al., 2010), it may be worthwhile to complement future community-based mPPIs with some economic components (e.g., asset transfers) to optimise the gains and the effects of both interventions. A research and policy implication of this finding is that poverty and mental health have strong associations and that it may be beyond the scope of any mono-component (i.e., mental health- or economic-focused) intervention to promote positive mental health in rural poor contexts.

Participants’ suggestions to include other health-related topics in the intervention programme, such as physical health, are important to consider for future community-based group mental health interventions. The call to complement the sessions with topics on physical health, in itself, is an indication of the need for a holistic, biopsychosocial approach to health and well-being that takes into consideration the physical and mental health aspects of an individual (see Keyes, 2014; Wilson Fadiji et al., 2019), and underscores the complementarities between mental and physical health. The physically demanding nature of farming with non-mechanised farm implements, compounded with the non-availability of healthcare facilities in the rural poor communities, necessitate a holistic approach to cater for the mental and physical health needs of the populace. Furthermore, while it is beyond the scope of this study to explore spillover effects of the ILP, participants’ recommendation to include spouses and friends (who showed keen interest in the sessions and homework assignments) could suggest a possible cross-over effects of community-based mental health interventions, particularly in collectivistic rural African settings.

Although the overarching aim was to develop an intervention that was appropriate for the current context, some findings from the current study may be transferrable to other contexts with similar characteristics. By facilitating inter-personal change processes through therapeutic interactions and member support, it is possible, for instance, for the programme to cause participants from similar socioeconomic and cultural settings to introspect, self-disclose (i.e., open up and share their lives and views with group members), and engage in a mutual trust and reciprocal commitment with one another. Furthermore, by encouraging participation in session discussions and homework assignments and by soliciting for feedback from each participant at sessions to evaluate their progress, it is possible for the programme to stimulate participants in similar contexts to cultivate a sense of responsibility and accountability to each other. It is also highly possible that participants from more collectivistic social settings, similar to the current context, could form strong social networks and relationships with group members by participating in the programme and engaging in group work. However, given that people differ in conceptualising, interpreting, and expressing well-being and happiness across cultures (see Tov & Nai, 2018), the findings describing participants’ perceived benefits of the programme in terms of promoting their well-being and happiness may be unique to the context of the current study. Similarly, considering that researchers solicited for and incorporated contributions of selected individuals from the study population (most of whom were peasant farmers) into the discussions and activities on goal-setting and personal growth and development, some discussions and examples in these sessions are focused on increasing vocational productivity among peasant farmers, which may only benefit participants in similar socioeconomic settings who are also peasant farmers.

### Study limitations

The current study has a few limitations. First, although participants were recruited from rural poor communities, they were all native Twi-speakers from one district and ethnic group in Ghana and thus an unrepresentative sample. Although findings from qualitative studies are usually not intended to be generalisable, the lack of diversity in the sample raises implications for the transferability of the findings to a socio-cultural context that is markedly different from that of this study. Second, the results from this qualitative study suggest a potential spillover effect of the intervention, considering that participants were required to discuss key aspects of the sessions and homework assignments with relatives or friends who were not part of the intervention programme. Future studies should consider building in and measuring the impact of learning transfer mechanisms beyond the homework assignments, such as peer support or group coaching by participants to non-participants in the communities.

## Conclusions

This study explored participants’ experiences of, perceived benefits of, and recommendations to improve the 10-week group-based mPPI (the inspired life programme; ILP) designed to promote positive mental health and reduce symptoms of depression and negative affect in a sample of rural poor adults in Ghana. The findings suggest that the ILP inspired the participants to introspect, practicalise and fructify their ideas and experiences, develop mutually beneficial partnerships, motivated participants to learn effective strategies to improve personal well-being and mental health, fostered stronger social support networks, and caused positive changes in participants thinking and behaviour that led to increased vocational productivity. The findings of this study may be relevant to mental health professionals and organisations working to promote the mental health of individuals living in rural poor settings. The results are also important from a practical perspective, in that group-level interventions, delivered at the community setting, can be cost-effective and can have positive effects on mental health state and positive functioning.

We conclude that the inclusion of qualitative study approaches in community-based participatory mental health research could facilitate our understanding of participants’ experiences and factors and processes that drive the therapeutic effects of mental health in resource-limited rural settings in sub-Saharan Africa. The qualitative approach also facilitated a deeper understanding of the further needs that may be catered for in future (multidisciplinary) programmes. The collectivistic orientation of the study context, the deprived socio-economic state of participants, the courteousness of session facilitators and their profound understanding of the study context and the principles and methods of ILP, as well as the community-based participatory approach used in the development and implementation of the ILP were key ingredients that influenced the findings of this study. The findings of this qualitative study help explain our previously reported quantitative findings that showed medium to large effects of the ILP on positive mental health with a corresponding decrease in the levels of depression and negative affect post-intervention and at follow-up in the intervention group, as compared to the control group (see Appiah, Wilson-Fadiji, et al., 2020).

## References

[cit0001] Abukari, Z. (2018). “Not Giving Up”: Ghanaian students’ perspectives on resilience, risk, and academic achievement. *SAGE Open*, 8(4), 21–14. 10.1177/2158244018820378

[cit0002] American Psychological Association, Presidential Task Force on Evidence-Based Practice. (2006). Evidence-based practice in psychology. *American Psychologist*, 61(4), 271–285. 10.1037/0003-066X.61.4.271

[cit0003] Appiah, R. (2020). Community-based participatory research in rural African contexts: Ethico-cultural considerations and lessons from Ghana. *Public Health Reviews*, 41(1), 27. 10.1186/s40985-020-00145-2 33292760 PMC7694909

[cit0004] Appiah, R., Schutte, L., Wilson Fadiji, A., Wissing, M. P., & Cromhout, A. (2020). Factorial validity of the Twi versions of five measures of mental health and well-being in Ghana. *PloS One*, 15(8), e0236707. 10.1371/journal.pone.0236707 32780773 PMC7418998

[cit0005] Appiah, R., Wilson-Fadiji, A., Schutte, L., & Wissing, M. P. (2020). Effects of a community-based multicomponent positive psychology intervention on mental health of rural adults in Ghana. *Applied Psychology: Health and Well-Being*, 12(3), 828–862. 10.1111/aphw.12212 32706933

[cit0006] Bach, J. M., & Guse, T. (2015). The effect of contemplation and meditation on ‘great compassion’ on the psychological well-being of adolescents. *Journal of Positive Psychology*, 10(4), 359–369. 10.1080/17439760.2014.965268

[cit0007] Beck, J. S. (2011). *Cognitive behavior therapy: Basics and beyond (2nd ed**.)*. New York: Guilford Press.

[cit0008] Bohlin, I., & Sager, M. (Eds.). (2011). *Evidensens många ansikten: evidensbaserad praktik i praktiken* [The many faces of evidence: A close look at evidence-based practice]. Arkiv.

[cit0009] Bolier, L., Haverman, M., Kramer, J., Westerhof, G. J., Riper, H., & Walburg, J. A. (2013). Positive psychology interventions: A meta-analysis of randomized controlled studies. *BMC Public Health*, *13,*119. 10.1186/1471-2458-13-119 PMC3599475 23390882

[cit0010] Borek, A. J., & Abraham, C. (2018). How do small groups promote behaviour change? An integrative conceptual review of explanatory mechanisms. *Applied Psychology: Health and Well-Being*, 10(1), 30–61. 10.1111/aphw.12120 29446250

[cit0011] Braun, V., & Clarke, V. (2012). Thematic analysis. In Cooper H. (Ed.), *APA Handbook of Research Methods in Psychology:* Vol. 2*. Research designs* (pp. 57–91) Washington, DC: American Psychological Association

[cit0012] Bruhn, A. L., Mcdaniel, S. C., Fernando, J., & Troughton, L. (2016). Goal-setting interventions for students with behavior problems: A systematic review. *Behavioral Disorders*, 41(2), 107–121. 10.17988/0198-7429-41.2.107

[cit0013] Chakhssi, F., Kraiss, J. T., Sommers‐Spijkerman, M., & Bohlmeijer, E. T. (2018). The effect of positive psychology interventions on well‐being and distress in clinical samples with psychiatric or somatic disorders: A systematic review and meta‐analysis. *BMC Psychiatry*, 18(1), 211. 10.1186/s12888-018-1739-2 29945603 PMC6020379

[cit0014] Clark, D., Gill, D., Prowse, V., & Rush, M. (2017). Using goals to motivate college students: Theory and evidence from field experiments. IZA Discussion Papers. No. 10283.

[cit0015] Cleo, G., Hersch, J., & Thomas, R. (2018). Participant experiences of two successful habit-based weight-loss interventions in Australia: A qualitative study. *BMJ Open*, 8(5), e020146. 10.1136/bmjopen-2017-020146 PMC5988089 29858412

[cit0016] Creswell, J. W. (2014). *Research design: Qualitative, quantitative and mixed methods approaches* (4th ed.). Sage. 10.7748/nr.12.1.82.s2 28718745

[cit0017] Cunha, L. F., Pellanda, L. C., & Reppold, C. T. (2019). Positive psychology and gratitude interventions: A randomized clinical trial. *Frontiers in Psychology*, 10, Article 584. 10.3389/fpsyg.2019.00584 PMC6437090 30949102

[cit0018] Curry, O. S., Rowland, L. A., Van Lissa, C. J., Zlotowitz, S., McAlaney, J., & Whitehouse, H. (2018). Happy to help? A systematic review and meta-analysis of the effects of performing acts of kindness on the well-being of the actor. *Journal of Experimental Social Psychology, 76*, 320–329. 10.1016/j.jesp.2018.02.014

[cit0019] Damreihani, N., Behzadipour, S., Haghpanh, S., & Bordbar, M. (2018). The effectiveness of positive psychology intervention on the well-being, meaning, and life satisfaction of mothers of children with cancer: A brief report. *Journal of Psychosocial Oncology*, 36(3), 382–388. 10.1080/07347332.2018.1427173 29558290

[cit0020] Darwish, A. F. E., & Huber, G. L. (2003). Individualism vs. collectivism in different cultures: A cross-cultural study. *Intercultural Education*, 14(1), 47–56. 10.1080/1467598032000044647

[cit0021] Deng, Y., Xiang, R., Zhu, Y., Li, Y., Yu, S., & Liu, X. (2019). Counting blessings and sharing gratitude in a Chinese prisoner sample: Effects of gratitude-based interventions on subjective well-being and aggression. *The Journal of Positive Psychology*, 14(3), 303–311. 10.1080/17439760.2018.1460687

[cit0022] Engel, G. L. (1977). The need for a new medical model: A challenge for biomedicine. *Science*, 196(4286), 129–136. 10.1126/science.847460 847460

[cit0023] Ghana Statistical Service, Ghana Health Service, & ICF International. (2015). *Ghana demographic and health survey 2014*. GSS, GHS, and ICF International.

[cit0024] Gil-Rivas, V., Handrup, C. T., Tanner, E., & Walker, D. K. (2019). Global mental health: A call to action. *American Journal of Orthopsychiatry*, 89(4), 420–425. 10.1037/ort0000373 31169390

[cit0025] Goerg, S. J. (2015). Goal setting and worker motivation. *IZA World of Labor*, 178, 1–10. 10.15185/izawol.178

[cit0026] Guba, E. G., & Lincoln, Y. (1989). *Fourth generation evaluation*. Sage.

[cit0027] Gyekye, K. (2013). *Philosophy culture and vision: African perspectives: Selected essays*. Sub-Saharan Publishers.

[cit0028] Hammarberg, K., Kirkman, M., & De Lacey, S. (2016). Qualitative research methods: When to use them and how to judge them. *Human Reproduction*, 31(3), 498–501. 10.1093/humrep/dev334 26759142

[cit0029] Hariton, E., & Locascio, J. J. (2018). Randomised controlled trials - the gold standard for effectiveness research: Study design: Randomised controlled trials. *BJOG: An International Journal of Obstetrics and Gynaecology*, 125(13), 1716. 10.1111/1471-0528.15199 29916205 PMC6235704

[cit0030] Hendriks, T., Hassankhan, A., Schotanus-Dijkstra, M., Graafsma, T., Bohlmeijer, E. T., & de Jong, J. (2018). The efficacy of non-Western positive psychology interventions: A meta-analysis and systematic review. *International Journal of Wellbeing*,8(1), 71–98.

[cit0031] Hendriks, T., Schotanus-Dijkstra, M., Hassankhan, A., De Jong, J., & Bohlmeijer, E. (2019). The efficacy of multi-component positive psychology interventions: A Systematic review and meta-analysis of randomized controlled trials. *Journal of Happiness Studies*, *21*(1), 357-390. 10.1007/s10902-019-00082-1

[cit0032] Hodges, S. D., Laurent, S. M., & Lewis, K. L. (2011).Specially motivated, feminine, or just female: Do women have an empathic accuracy advantage? In J. L. Ickes Smith, W., Hall J., & Hodges S. D. (Eds.), *Managing interpersonal sensitivity: Knowing when—and when not—to understand others* (pp. 59–73). New York : Nova Science Publishers.

[cit0033] Ilvig, P. M., Kjær, M., Jones, D., Christensen, J. R., & Andersen, L. N. (2018). What an eye-opener” – A qualitative study of vulnerable citizens participating in a municipality-based intervention. *International Journal of Qualitative Studies on Health and Well-being*, 13(1), 1438698. 10.1080/17482631.2018.1438698 29488443 PMC5844043

[cit0034] Jin, D., & Nida, E. A. (2006). *On translation: An expanded edition*. City University of Hong Kong Press.

[cit0035] Kaplan, S., Bradley-Geist, J. C., Ahmad, A., Anderson, A., Hargrove, A. K., & Lindsey, A. (2014). A test of two positive psychology interventions to increase employee well-being. *Journal of Business and Psychology*, 29(3), 367–380. 10.1007/s10869-013-9319-4

[cit0036] Kashdan, T. B., Biswas-Diener, R., & King, L. A. (2008). Reconsidering happiness: The costs of distinguishing between hedonics and eudaimonia. *Journal of Positive Psychology*, 3(4), 219–233. 10.1080/17439760802303044

[cit0037] Kerkelä, E. S., Jonsson, L., Lindwall, M., & Strand, J. (2015). Individual experiences following a 6-month exercise intervention: A qualitative study. *International Journal of Qualitative Studies on Health and Well-being*, 10(1), 26376. 10.3402/qhw.v10.26376 26282865 PMC4539384

[cit0038] Keyes, C. L. (2014). Mental health as a complete state: How the salutogenic perspective completes the picture. In Bauer G. F. & Hamming O. (Eds.), *Bridging occupational, organizational and public health* (pp. 179–192). Dordrecht, The Netherlands: Springer.

[cit0039] Keyes, C. L. M. (2005). Mental illness and/or mental health? Investigating axioms of the complete state model of health. *Journal of Consulting and Clinical Psychology*, *73*(4), 539–548. 10.1037/0022-006X.73.3.539 15982151

[cit0040] Keyes, C. L. M., & Martin, C. C. (2017). The complete state model of mental health. In M. Slade, L. Oades, & A. Jarden (Eds.), *Wellbeing, recovery and mental health* (pp. 86–97). Cambridge University Press.

[cit0041] Koch, T. (1994). Establishing rigour in qualitative research: The decision trail. *Journal of Advanced Nursing*, 19(5), 976–986. 10.1111/j.1365-2648.1994.tb01177.x 8056928

[cit0042] Kpanake, L. (2018). Cultural concepts of the person and mental health in Africa. *Transcultural Psychiatry*, 55(2), 198–218. 10.1177/1363461517749435 29400136

[cit0043] Lally, P., Wardle, J., & Gardner, B. (2011). Experiences of habit formation: A qualitative study. *Psychology, Health & Medicine*, 16(4), 484–489. 10.1080/13548506.2011.555774 21749245

[cit0044] Levitt, H. M., Motulsky, S. L., Wertz, F. J., Morrow, S. L., & Ponterotto, J. G. (2017). Recommendations for designing and reviewing qualitative research in psychology: Promoting methodological integrity. *Qualitative Psychology*, 4(1), 2–22. 10.1037/qup0000082

[cit0045] Lincoln, Y., & Guba, E. G. (1985). *Naturalistic inquiry*. Sage. https://uk.sagepub.com/en-gb/afr/naturalistic-inquiry/book842

[cit0046] Lyubomirsky, S., & Layous, K. (2013). How do simple positive activities increase well-being? *Current Directions in Psychological Science*, 22(1), 57–62. 10.1177/0963721412469809

[cit0047] Martela, F., & Steger, M. F. (2016). The three meanings of meaning in life: Distinguishing coherence, purpose, and significance. *The Journal of Positive Psychology,* 11(5), 531–545. 10.1080/17439760.2015

[cit0048] McArdle, S., McGale, N., & Gaffney, P. (2012). A qualitative exploration of men’s experiences of an integrated exercise/CBT mental health promotion programme. *International Journal of Men’s Health*, 11(3), 240–257. 10.3149/jmh.1103.240

[cit0049] Mitchell, F., Stalker, K., Matthews, L., Mutrie, N., Melling, C., McConnachie, A., Murray, H., & Melville, C. A. (2018). A qualitative exploration of participants’ experiences of taking part in a walking programme: Perceived benefits, barriers, choices and use of intervention resources. *Journal of Applied Research in Intellectual Disabilities*, 31(1), 110–121. 10.1111/jar.12326 28004473

[cit0050] Moxham, L., Taylor, E. K., Patterson, C., Perlman, D., Brighton, R., Heffernan, T., & Sumskis, S. (2017). Goal setting among people living with mental illness: A qualitative analysis of recovery camp. *Issues in Mental Health Nursing*, 38(5), 420–424. 10.1080/01612840.2016.1271067 28165874

[cit0051] Odou, N., & Vella-Brodrick, D. A. (2013). The efficacy of positive psychology interventions to increase well-being and the role of mental imagery ability. *Social Indicators Research*, 110(1), 111–129. 10.1007/s11205-011-9919-1

[cit0052] Page, K. M., & Vella-Brodrick, D. A. (2013). The working for wellness program: RCT of an employee well-being intervention. *Journal of Happiness Studies*, 14(3), 1007–1031. 10.1007/s10902-012-9366-y

[cit0053] Patel, V., Weiss, H. A., Chowdhary, N., Naik, S., Pednekar, S., Chatterjee, S., & Kirkwood, B. R. (2010). Effectiveness of an intervention led by lay health counsellors for depressive and anxiety disorders in primary care in Goa, India (MANAS): A cluster randomised controlled trial. *Lancet*,376 (9758), 2086–2095. 10.1016/S0140-6736(10)61508-5 PMC4964905 21159375

[cit0054] Pietrowsky, R., & Mikutta, J. (2012). Effects of positive psychology interventions in depressive patients—A randomized control study. *Psychology*, 3(12), 1067–1073. 10.4236/psych.2012.312158

[cit0055] Pretorius, C., Venter, C., Temane, M., & Wissing, M. (2008). The design and evaluation of a hope enhancement programme for adults. *Journal of Psychology in Africa*, 18(2), 301–308. 10.1080/14330237.2008.10820202

[cit0056] Prilleltensky, I. (2019). Mattering at the intersection of psychology, philosophy and politics. American. *Journal of Community Psychology*, 65(1–2), 16–34. 10.1002/ajcp.12368 31407358

[cit0057] Proyer, R. T., Wellenzohn, S., Gander, F., & Ruch, W. (2015). Toward a better understanding of what makes positive psychology interventions work: predicting happiness and depression from the person × intervention fit in a follow-up after 3.5 years. *Applied psychology Health and well-being,* 7(1), 108–128. 10.1111/aphw.12039 25424973

[cit0058] Rippstein-Leuenberger, K., Mauthner, O., Bryan Sexton, J., & Schwendimann, R. (2017). A qualitative analysis of the Three Good Things intervention in healthcare workers. *BMJ Open*, 7(5), e015826. 10.1136/bmjopen-2017-015826 PMC5623381 28611090

[cit0059] Rowland, L., & Curry, O. S. (2018). A range of kindness activities boost happiness. *Journal of Social Psychology*, 159(3), 340–343. 10.1080/00224545.2018.1469461 29702043

[cit0060] Ruini, C., & Ryff, C. D. (2016). Using eudaimonic well-being to improve lives. In Wood A. M. & Johnson J. (Eds.), *The Wiley handbook of positive clinical psychology* (p. 153–166). Wiley Blackwell. 10.1002/9781118468197.ch11

[cit0061] Ryff, C. D. (2014). Psychological well-being revisited: Advances in the science and practice. *Psychotherapy and Psychosomatics*, 83(1), 10–28. 10.1159/000353263 24281296 PMC4241300

[cit0062] Sawka, K. J., McCormack, G. R., Nettel-Aguirre, A., Hawe, P., & Doyle-Baker, P. K. (2013). Friendship networks and physical activity and sedentary behavior among youth: A systematized review. *The International Journal of Behavioral Nutrition and Physical Activity*, 10(1), 130. 10.1186/1479-5868-10-130 24289113 PMC4220781

[cit0063] Schotanus-Dijkstra, M., Pieterse, M. E., Drossaert, C. H. C., Walburg, J. A., & Bohlmeijer, E. T. (2019). Possible mechanisms in a multicomponent email guided positive psychology intervention to improve mental well-being, anxiety and depression: A multiple mediation model. *Journal of Positive Psychology*, 14(2), 141–155. 10.1080/17439760.2017.1388430

[cit0064] Sin, N. L., & Lyubomirsky, S. (2009). Enhancing well-being and alleviating depressive symptoms with positive psychology interventions: A practice-friendly meta-analysis. *Journal of Clinical Psychology*, 65(5), 467–487. 10.1002/jclp.20593 19301241

[cit0065] Skeen, S., Lund, C., Kleintjes, S., Flisher, A., & The MHaPP Research Programme Consortium. (2010). Meeting the Millennium Development Goals in Sub-Saharan Africa: What about mental health? *International Review of Psychiatry*, 22(6), 624–631. 10.3109/09540261.2010.535509 21226650

[cit0066] Smith, J. L., & Hanni, A. A. (2019). Effects of a savoring intervention on resilience and well-being of older adults. *Journal of Applied Gerontology*, 38(1), 137–152. 10.1177/0733464817693375 28380722

[cit0067] Spieth, P. M., Kubasch, A. S., Penzlin, A. I., Illigens, B. M., Barlinn, K., & Siepmann, T. (2016). Randomized controlled trials - a matter of design. *Neuropsychiatric Disease and Treatment*, 12, 1341–1349. 10.2147/NDT.S101938 27354804 PMC4910682

[cit0068] Teodorczuk, K., Guse, T., & Du Plessis, G. A. (2019). The effect of positive psychology interventions on hope and well-being of adolescents living in a child and youth care centre. *British Journal of Guidance & Counselling*, 47(2), 234–245. 10.1080/03069885.2018.1504880

[cit0069] Tong, A., Sainsbury, P., & Craig, J. (2007). Consolidated criteria for reporting qualitative research (COREQ): A 32-item checklist for interviews and focus groups. *International Journal for Quality in Health Care*, 19(6), 349–357. 10.1093/intqhc/mzm042 17872937

[cit0070] Tough, H., Siegrist, J., & Fekete, C. (2017). Social relationships, mental health and wellbeing in physical disability: A systematic review. *BMC Public Health*, 17(1), 414. 10.1186/s12889-017-4308-6 28482878 PMC5422915

[cit0071] Tov, W., & Nai, Z. L. S. (2018). Cultural differences in subjective well-being: How and why. In J. E. Maddux (Ed.), *Frontiers of social psychology. Subjective well-being and life satisfaction* (pp. 50–73). Routledge/Taylor & Francis Group.

[cit0072] Van Woerkom, M., & Meyers, M. C. (2019). Strengthening personal growth: The effects of a strengths intervention on personal growth initiative. *Journal of Occupational and Organisational Psychology*, 92(1), 98–121. 10.1111/joop.12240

[cit0073] Van Zyl, L. E., & Rothmann, S. (2012). Beyond smiling: The evaluation of a positive psychological intervention aimed at student happiness. *Journal of Psychology in Africa*, 22(3), 369–384. 10.1080/14330237.2012.10820541

[cit0074] Weiss, L. A., Westerhof, G. J., & Bohlmeijer, E. T. (2016). Can we increase psychological well-being? The effects of interventions on psychological well-being: A meta-analysis of randomised controlled trials. *PLoS One*, *11*(6), e0158092. 10.1371/journal.pone.0158092 27328124 PMC4915721

[cit0075] Westerhof, G., & Keyes, C. L. M. (2010). Mental illness and mental health: The two continua model across the lifespan. *Journal of Adult Development*, 17(2), 110–119. 10.1007/s10804-009-9082-y 20502508 PMC2866965

[cit0076] Wilson Fadiji, A., Meiring, L., & Wissing, M. P. (2019). Understanding well-being in the Ghanaian context: Linkages between lay conceptions of well-being and measures of hedonic and eudaimonic well-being. *Applied Research in Quality of Life*, Advance online publication. 10.1007/s11482-019-09777-2

[cit0077] Wissing, M. P., & Temane, Q. M. (2013). The prevalence of levels of wellbeing revisited in an African context. In C. L. M. Keyes (Ed.), *Mental well-being: International contributions to the study of positive mental health* (Chapter 4, pp. 71–90). Springer.

